# Supplementary material for: Congenital Aneuploidy in Klinefelter Syndrome with B-Cell Acute Lymphoblastic Leukemia Might Be Associated with Chromosomal Instability and Reduced Telomere Length
Source: Cancers (Basel). 2022 May 6;14(9):2316. doi: 10.3390/cancers14092316 (PMC9136641; doi:10.3390/cancers14092316)
Supplement: Supplementary file 1 [file cancers-14-02316-s001.zip › cancers-1682029-supplementary.pdf]

Supplementary material

Table S1: Case reports of acute lymphoblastic leukemia associated with Klinefelter syndrome

Year	Age	Diagnosis	PB	Diagnostic BM: Conventional Cytogenetics	Ref
1963	32 yrs	ALL	47,XXYc	not done	[1]
1966	n.a.	ALL	47,XXYc	not done	[2]
1974	19 yrs	ALL	48,XXXYc	not done	[3]
1984	9 days	ALL-L2	47,XXYc	not done	[4]
1990	n.a.	ALL	-	47,XXYc,del(7)(q22),add(19)(p?),-20,+mar[19]/47,XXYc[2]	[5]
1992	21 months	ALL	47,XXYc	47,XXYc	[6]
	3.5 yrs	ALL	46,XY/47,XXYc	46,XY/47,XXYc	do
1994	4 yrs	ALL-L2	47,XXYc	47,XXYc	[7]
1999	n.a.	ALL	-	46,XXYc,del(7)(q22),add(19)(p13),-20[19]/47,XXYc[2]	[8]
	49 yrs	ALL-L2	46,XY/47,XXYc	47,XXYc,t(9;22;11)(q34;q11;q13)[10]/46,XY[6]/47,XXY[4]	[9]
2002	2.5 yrs	ALL-L1	47,XXYc	54,XXYc,+4,+8,+9,+12,+17,+18,+21[3]/47,XXY[16]	[10]
2004	17 yrs	B-ALL	47,XXYc[1]/48,XXXY[9]	47,XXYc[5]/46,XY[8]	[11]
2008	3.9	B-ALL	-	54,XXYc,+4,+8,+9,+14,+16,+18,+21[cp6]/46,XY[28](47,XXYc[18]	[12]
2016	14 yrs	B-ALL	-	47,XXYc,dic(7;16)(p11;p13),+21[7]	[13]
2016	n.a.	B-ALL	-	47,XXYc[21]	[14]
	n.a.	B-ALL	-	47,XXYc[20]	do
	n.a.	B-ALL	-	47,XY,del(X)(q24),del(6)(q13q21),-19,+mar[3]/47,XXYc	do
	n.a.	B-ALL	-	47,XXYc,der(11)t(11;12)(q13;p13),der(12)t(11;12)(q23;p13)ins(12;11)(q24;q13q23), der(15)t(8;15)(q13;p13)[7]/47,XXYc[4]	do

	n.a.	B-ALL	-	61,XXYc,+X,+4,+4,del(6)(q21),+6,+10,+11,+14,+17,+18,+19,+20,+21,+21+mar[cp11]/47,XXYc[9]	do
	n.a.	B-ALL	47,XXYc	47,XXYc,t(1;19)(q23;p13.3)[5]/47,XXYc,der(19)t(1;19)(q23;p13.3)[6]/47,XXYc[6]	do
	n.a.	B-ALL	47,XXYc	46,XY,-X,inv(4)(p15.2p16),del(9)(p13p12)[12]/45,idem,-7[7]/47,XXYc[21]	do
	n.a.	B-ALL	47,XXYc	58,XXYc,+4,+6,+8,+10,+11,+12,+14,+18,+19,add(19)(p13.3),+21,+21[cp13]/47,XXYc[13]	do
	n.a.	B-ALL	47,XXYc	50,XXYc,+X,+20,+21[13]/47,XXYc[7]	do
	n.a.	B-ALL	-	55,XXYc,+X,+X,dup(1)(q12q32),+4,+6,i(7)(q10),+11,+18,+21,+21[14]/47,XXYc[6]	do
	n.a.	B-ALL	47,XXYc	not done	do
2022	3 yrs	pre-B ALL	47,XXYc	47,XXYc,del(7)(p13)[2]/47,idem,+10,-15[4]/47,XXYc[19]	Present case

n.a.: not available. PB: peripheral blood; BM: bone marrow

Supplementay references.

1. Bousser, J.; Tanzer, J. [Klinefelter syndrome and acute leukemia; apropos of a case]. Nouv Rev Fr Hematol 1963, 3, 194-197.
2. Ruffie, J.; Colombies, P.; Combes, P.F.; Ducos, J. [Lymphoblastic leukemia in a patient with complex congenital anomaly (probable type XXY)]. Bull Acad Natl Med 1966, 150, 342-346.
3. Sohn, K.Y.; Boggs, D.R. Klinefelter's syndrome, LSD usage and acute lymphoblastic leukemia. Clin Genet 1974, 6, 20-22, doi:10.1111/j.1399-0004.1974.tb00625.x.
4. Gale, G.B.; Toledano, S.R. Congenital acute lymphocytic leukemia in a newborn with Klinefelter syndrome. Am J Pediatr Hematol Oncol 1984, 6, 338-339, doi:10.1097/00043426-198423000-00020.
5. Walters, R.; Kantarjian, H.M.; Keating, M.J.; Estey, E.H.; Trujillo, J.; Cork, A.; McCredie, K.B.; Freireich, E.J. The importance of cytogenetic studies in adult acute lymphocytic leukemia. Am J Med 1990, 89, 579-587, doi:10.1016/0002-9343(90)90175-d.
6. Shaw, M.P.; Eden, O.B.; Grace, E.; Ellis, P.M. Acute lymphoblastic leukemia and Klinefelter's syndrome. Pediatr Hematol Oncol 1992, 9, 81-85, doi:10.3109/08880019209006400.
7. Gurgey, A.; Kara, A.; Tuncer, M.; Alikasifoglu, M.; Tuncbilek, E. Acute lymphoblastic leukemia associated with Klinefelter syndrome. Pediatr Hematol Oncol 1994, 11, 227-229, doi:10.3109/08880019409141662.
8. Dabaja, B.S.; Faderl, S.; Thomas, D.; Cortes, J.; O'Brien, S.; Nasr, F.; Pierce, S.; Hayes, K.; Glassman, A.; Keating, M.; et al. Deletions and losses in chromosomes 5 or 7 in adult acute lymphocytic leukemia: incidence, associations and implications. Leukemia 1999, 13, 869-872, doi:10.1038/sj.leu.2401430.
9. Yano, T.; Yuzurio, S.; Kimura, K.; Kshimoto, T. Ph-positive acute lymphocytic leukemia in a man with Klinefelter syndrome. Cancer Genet Cytogenet 2000, 118, 83-84, doi:10.1016/s0165-4608(99)00154-5.

10. Keung, Y.K.; Buss, D.; Chauvenet, A.; Pettenati, M. Hematologic malignancies and Klinefelter syndrome. a chance association? Cancer Genet Cytogenet 2002, 139, 9-13, doi:10.1016/s0165-4608(02)00626-x.
11. Machatschek, J.N.; Schrauder, A.; Helm, F.; Schrappe, M.; Claviez, A. Acute lymphoblastic leukemia and Klinefelter syndrome in children: two cases and review of the literature. Pediatr Hematol Oncol 2004, 21, 621-626, doi:10.1080/08880010490501024.
12. Sharathkumar, A.; DeCamillo, D.; Bhambhani, K.; Cushing, B.; Thomas, R.; Mohamed, A.N.; Ravindranath, Y.; Taub, J.W. Children with hyperdiploid but not triple trisomy (+4,+10,+17) acute lymphoblastic leukemia have an increased incidence of extramedullary relapse on current therapies: a single institution experience. Am J Hematol 2008, 83, 34-40, doi:10.1002/ajh.21011.
13. Ivanov Ofverholm, I.; Tran, A.N.; Olsson, L.; Zachariadis, V.; Heyman, M.; Rudd, E.; Syk Lundberg, E.; Nordenskjold, M.; Johansson, B.; Nordgren, A.; et al. Detailed gene dose analysis reveals recurrent focal gene deletions in pediatric B-cell precursor acute lymphoblastic leukemia. Leuk Lymphoma 2016, 57, 2161-2170, doi:10.3109/10428194.2015.1136740.
14. Rau, R.E.; Carroll, A.J.; Heerema, N.A.; Arland, L.; Carroll, W.L.; Winick, N.J.; Raetz, E.A.; Loh, M.L.; Yang, W.; Relling, M.V.; et al. Klinefelter syndrome and 47,XYY syndrome in children with B cell acute lymphoblastic leukaemia. Br J Haematol 2017, 179, 843-846, doi:10.1111/bjh.14258.
